# Effect of selected biocides on microbiologically influenced corrosion caused by *Desulfovibrio ferrophilus* IS5

**DOI:** 10.1038/s41598-018-34789-7

**Published:** 2018-11-09

**Authors:** Mohita Sharma, Hongwei Liu, Shiqiang Chen, Frank Cheng, Gerrit Voordouw, Lisa Gieg

**Affiliations:** 1 Petroleum Microbiology Research Group, Department of Biological Sciences, University of Calgary, Calgary, Alberta T2N 1N4 Canada; 2 Department of Mechanical and Manufacturing Engineering, University of Calgary, Calgary, Alberta T2N 1N4 Canada; 3 Present Address: Institute of Marine Science and Technology, Shandong University, 72 Binhai Road, Qingdao, 266237 P. R. China

## Abstract

The marine bacterial strain *Desulfovibrio ferrophilus* IS5, known for its lithotrophic growth ability to use metallic iron as a sole electron donor and for causing corrosion of steel, was used in the current study. Four commonly used biocides in the oil and gas industry, namely tetrakis(hydroxymethyl) phosphonium sulfate (THPS), glutaraldehyde (GLUT), benzalkonium chloride (BAC), and GLUT/BAC were selected to study their efficacy in controlling carbon steel corrosion in the presence of this strain. Incubations containing strain IS5 and low carbon steel coupons were prepared in the presence and absence of the four biocides, and these were monitored using both electrochemical methods (electrochemical impedance spectroscopy, linear polarization resistance and potentiodynamic polarization) and surface analyses (scanning electron microscopy, confocal measurements, optical microscopy, and profilometry) to assess the biofilm/metal interactions. When THPS, BAC, and GLUT/BAC treatments were applied, minimal corrosion was measured by all methods. In contrast, severe pitting was observed in the presence of 50 ppm GLUT, similar to what was observed when *D. ferrophilus* IS5 was incubated in the absence of biocide, suggesting that GLUT alone may not be effective in controlling MIC in marine environments. This study also showed that the use of non-destructive electrochemical methods is effective for screening for real time biocide selection and monitoring of the impact of chemicals post-dosage in oil and gas operations.

## Introduction

Low carbon (LC) steel is a commonly used material for the fabrication of metal infrastructure in marine offshore oil production applications as it is relatively inexpensive, easily manufactured, and readily available. However, LC steel is also susceptible to corrosion especially microbiologically influenced corrosion (MIC)^1–6^. Microorganisms have been reported to participate in localized attack on the metal infrastructure in seawater and other environments by enhancing pitting, dealloying, galvanic corrosion, hydrogen embrittlement, erosion corrosion, and stress corrosion cracking processes^7^. Seawater is also used as a coolant in heat exchangers in various industrial service water systems and shipping industries. The presence of microorganisms, especially sulfate-reducing microorganisms (SRM), have proven detrimental to the infrastructure and operation of these industries^8^.

Microbiologically influenced corrosion in high salinity environments is a serious environmental hazard and extensive research has been conducted towards effective MIC mitigation. SRM thrive in seawater, as it is naturally rich in sulfate (the electron acceptor for SRM), and have been extensively held responsible for localized corrosion or pitting corrosion of metal^9^. SRM able to directly scavenge electrons from a metal surface have been reported to be involved in carbon steel corrosion mechanisms, especially when nutrients are limited^10–13^. Seawater represents such a nutrient-limited environment where metal like LC steel could be the only potential electron donor source with sulfate present as electron acceptor, allowing for electroactive biofilms to thrive because of the corrosion potential of carbon steel in a marine environment^14^.

In fact, microorganisms capable of directly using metallic iron as an electron donor and aggressively attacking the metal infrastructure have been isolated from marine environments^10,11,15^. Such a mechanism has been termed electrical MIC, or EMIC, and has been reported to occur by SRM, methanogens, nitrate-reducers, and acetogens^14,16–22^. Some SRM have been known to aggressively attack carbon steel in the absence of an organic carbon source^18,22^. For example, *Desulfovibrio ferrophilus* strain IS5 is reported to be one such lithotrophic organism isolated from a seawater environment that is capable of an EMIC mechanism^11,23^. Electrical MIC processes have been reported to yield higher corrosion rates of steel (up to 0.7 mm/yr) compared to other MIC mechanisms^11,24^.

Curtailing microbiological growth in oil and gas systems is typically done via physical protection of the metal infrastructure using paints and coatings, pigging, and by treatment with chemicals like biocides and corrosion inhibitors. Understanding how these chemicals interact with the biofilm-metal interface of the metal infrastructure is important to ensure their efficacy in preventing corrosion. Hence, in the current study, the electrochemical behavior of carbon steel coupons in artificial sea water medium inoculated with the corrosive bacterial strain *Desulfovibrio ferrophilus* IS5 was studied in the presence and absence of four biocides commonly used in the oil and gas industry (Table 1) in order to understand the biofilm-electrode-chemical interface reactions using electrochemical and microscopic methods.

**Table 1:** Biocides used for this study, including their type and mode of action for inhibiting microorganisms.

Name	Type	Mode of action	References
Tetrakis(hydroxymethyl) phosphonium sulfate (THPS)	Quarternary phosphonium compound	Releases THP (trishydoxymethyl phosphine) which cleaves sulfur-sulfur bonds in the disulfide amino acids of microbial cell walls; damages cell membrane; removes iron sulfide deposits from the metal surface	^34, 36– 38^
Glutaraldehyde (GLUT)	Electrophilic	Contains reactive electron accepting functional groups that react with exposed amine thiol groups in membrane proteins of bacteria; potent crosslinker for amino acids; causes cell wall damage and cytoplasmic coagulation	^38, 41, 42^
Benzalkonium chloride (BAC)	Lytic (membrane active)	Amphipathic in nature, surfactants that can penetrate bacterial cell wall and lyse the cells resulting in the loss of osmoregulation capacity	^32, 38, 39^
GLUT/BAC (50/50)	Lytic + electrophilic	Combined actions of GLUT and BAC as they perform synergistically together	^38, 47^

## Results

**Figure 1:**
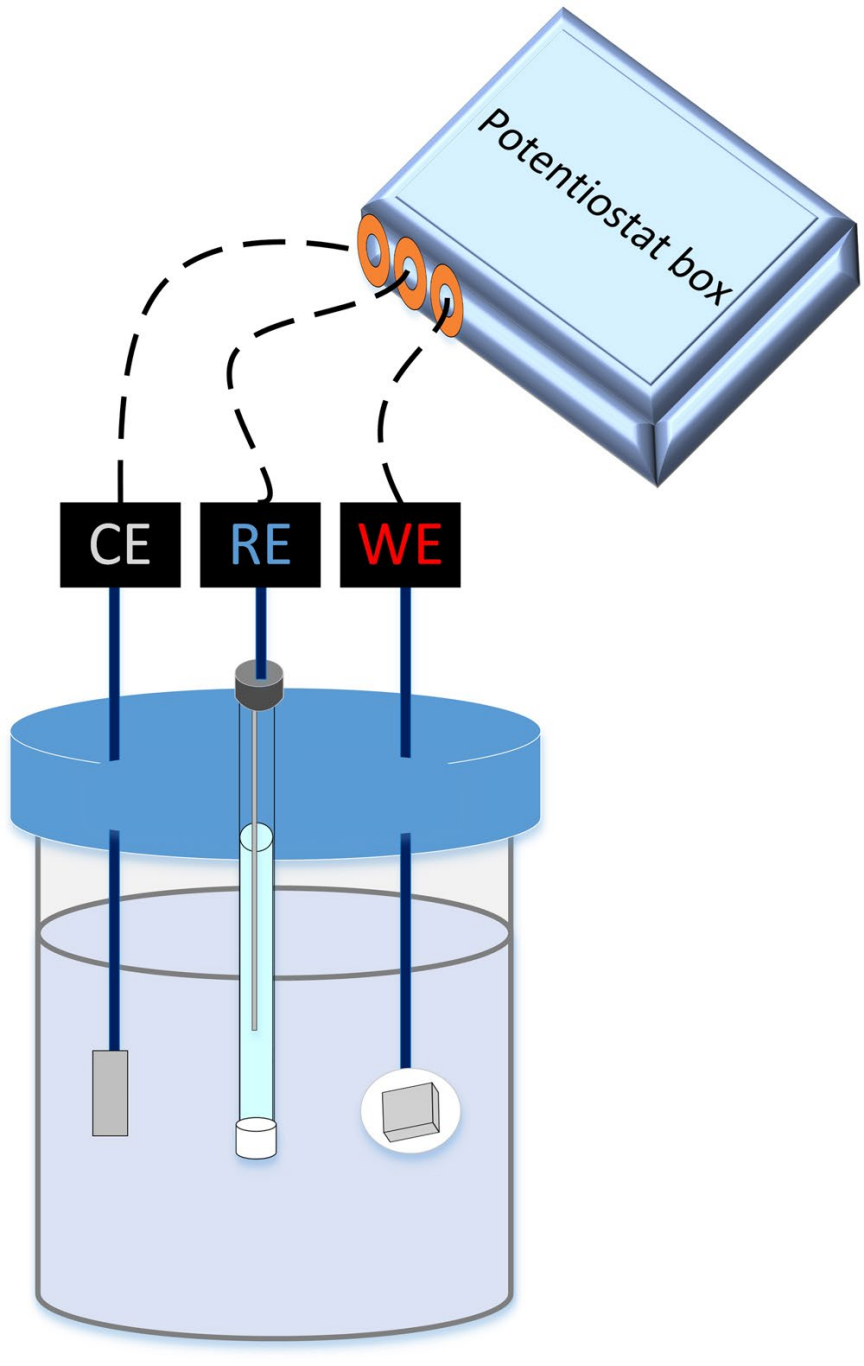
Schematic of the electrochemical cell configuration used for the experimental incubations in a N_2_:CO_2_ (80:20) atmosphere where platinum wire was used as the CE: counter electrode, LC steel coupon was used as the WE: working electrode, and Ag/AgCl/sat KCl was used as the RE: reference electrode.

In order to study the efficacy of commonly used biocides used in the oil and gas industry, a highly corrosive strain, *D. ferrophilus* IS5, was selected for performing MIC related electrochemical tests over a 15-d incubation period (Fig. 1). After 3 days of incubation, when 10 mM formate was utilized for the reduction of 2.5 mM sulfate and the remaining sulfate (~2.5 out of the initial 5 mM) was still available for the strain to get further reduced using iron as electron donor (determined using separate batch incubations), the cells were exposed to a 50 ppm dosage of one of 4 different biocides and monitored for another 12 days using electrochemical techniques, along with microscopic techniques at the end of the experiment.

### Surface analysis

**Figure 2:**
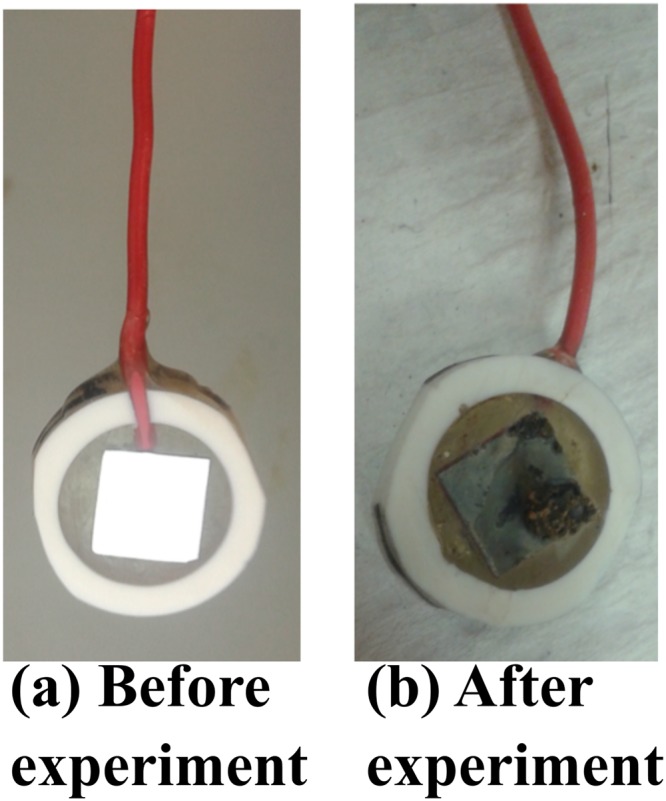
The surface of a coupon (**a**) before and (**b**) after the end of the 15-d experiment with ASW medium and *D. ferrophilus* IS5; no biocide was present in this experiment. In (**b**), note the mineral crust deposited at the coupon surface.

Once the experiment was terminated after the 15-d incubation period, a mineral crust was observed in samples incubated with IS5 in the absence of biocide (Fig. 2). *D. ferrophilus* IS5 has been previously reported to form a conductive mineral crust on the surface of the metal that showed electrical conductivity^11,15,20^. Hence, the formation of such a crust acted as a positive control for optimal growth of *D. ferrophilus* IS5 in ASW medium where no biocide was added during the course of the experiment.

**Figure 3:**
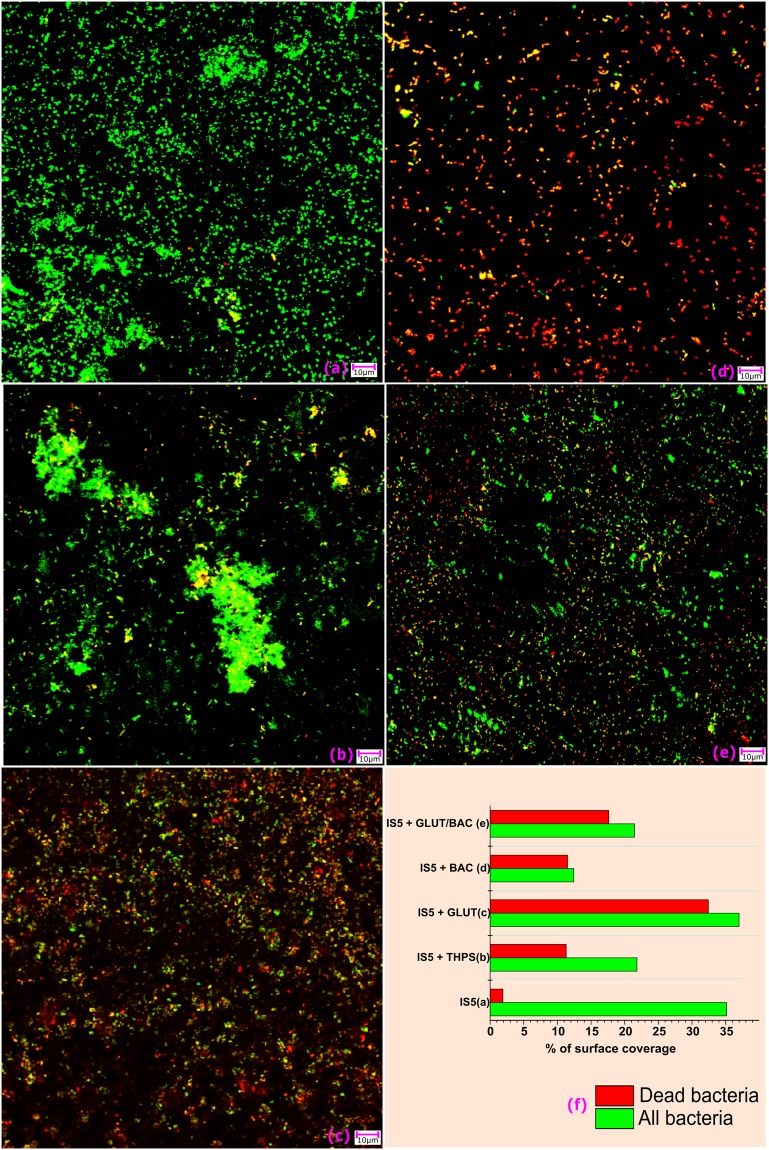
Confocal images of all the samples incubated under different conditions. (**a**) Positive control, IS5 only; (**b**) IS5 + THPS; (**c**) IS5 + GLUT; (**d**) IS5 + BAC; and (**e**) IS5 + GLUT/BAC_._ These images were taken at the end of the 15-day incubation. The graph in (**f**) shows the number of bacteria stained during the processing of these coupons (All/green stain) vs the number of damaged/dead cells identified using the counter stain (Dead/red stain). These values were calculated after statistical evaluation of multiple images/samples using the Gwyddion software.

The biofilm coverage on the surfaces of the coupons was also analyzed using confocal microscopy after 15 days of incubation (Fig. 3). The biofilm coverage was sparse in samples injected with biocide (Fig. 3b–e) as compared to the IS5 sample where no biocide was added (Fig. 3a,f summarizes the statistical results obtained from the comparison of different biocides from the confocal images). The LC steel coupon which consisted of only the IS5 strain (Fig. 3a) showed 35% surface coverage with biofilm out of which less than 5% were cells with damaged membranes or dead. A similar percentage of surface coverage was observed on the coupon where GLUT was added as a biocide (Fig. 3c). Though the surface coverage here was over 35%, most of the cells present in the biofilm were damaged, presumably due to the biocidal activity of GLUT. In the incubations with GLUT/BAC and THPS, less than 25% of area was covered with biofilm, out of which approximately 25% and 15% of the cell population were damaged, respectively. The least amount of biofilm (approximately 12%) was formed in incubations dosed with BAC and almost all of this population was already damaged or dead (Fig. 3d). This suggests that BAC was the most effective biocide in preventing formation of biofilm, or that it promoted the dissolution of biofilm from the surface of the LC steel coupon. BAC additionally controlled the remaining population present on the surface, at least under the experimental conditions tested here.

**Figure 4:**
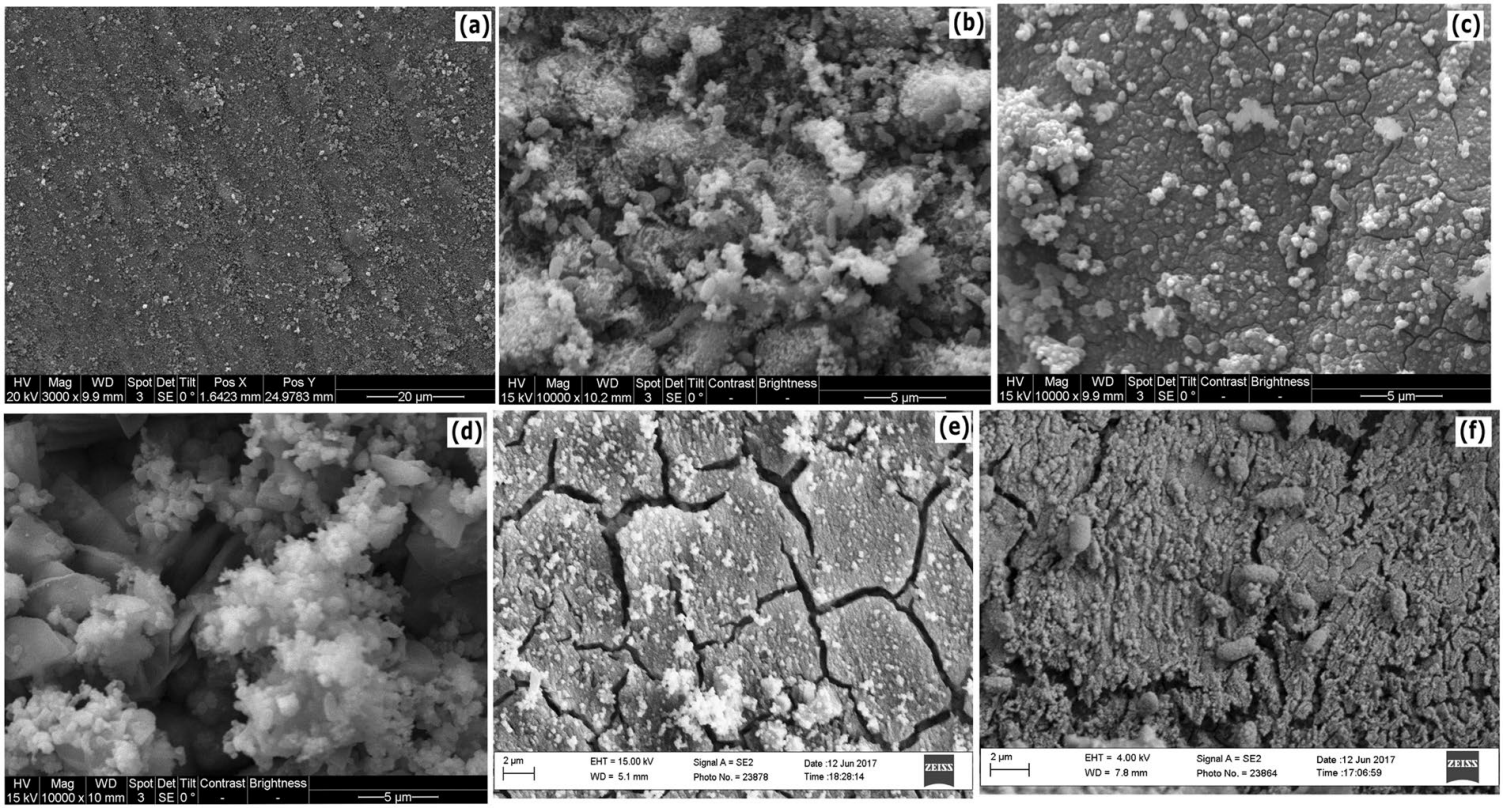
Scanning electron micrographs of all the low carbon steel coupons cleaned and visualized at the end of the 15-d incubation where (**a**) Sterile control (ASW medium only); (**b**) Positive control (ASW medium + IS5); (**c**) IS5 + THPS; (**d**) IS5 + GLUT; (**e**) IS5 + BAC; and (**f**) IS5 + GLUT/BAC.

Little corrosion product or film was seen in the scanning electron micrographs of the coupon retrieved from the sterile control incubation after 15 days (Fig. 4a). However, biofilm and cells as well as corrosion product formation were seen in the *D. ferrophilus* IS5 only incubation, IS5 with GLUT, and IS5 with GLUT/BAC after 15 days of incubation (Fig. 4b,d,f respectively). However, the amount of corrosion product deposit and biofilms were comparatively less in samples incubated with THPS (Fig. 4c) or BAC (Fig. 4e), corroborating the confocal micrograph results. These micrographs were also used for the qualitative evaluation of elemental composition of these films (based on weight %) using EDX (Figure S1). Elevated levels of O, P and Na were found in samples incubated with IS5 only and the IS5 and GLUT combination, as compared to other incubations. The signals corresponding to C, O, S, and P were higher in samples with IS5 than in the sterile control as these elements form an integral component of the biofilm and the corrosion deposit products such as FeCO_3_. However, further investigations would be required to confirm the presence of these products on the surface.

**Figure 5:**
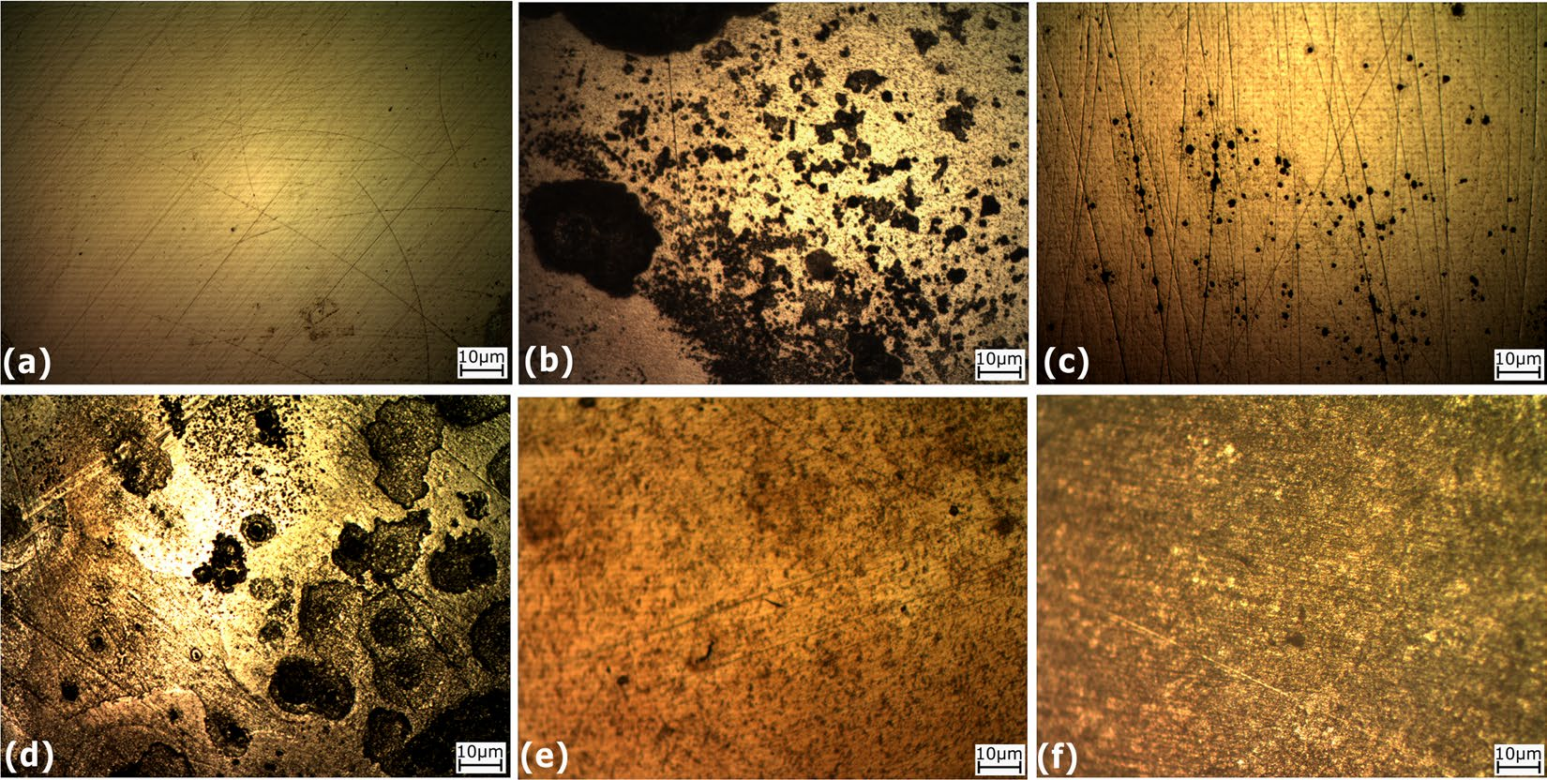
Optical micrographs of all the low carbon steel coupons cleaned and visualized at the end of 15 days where (**a**) Sterile control (ASW medium only); (**b**) Positive control (ASW medium + IS5); (**c**) IS5 + THPS; (**d**) IS5 + GLUT; (**e**) IS5 + BAC; and (**f**) IS5 + GLUT/BAC.

At the start of the experiment, the coupons were mirror polished and were very smooth without any defects (Fig. 2). Following the 15-d incubation, the sterile control coupon (incubated only with ASW medium) showed slight uniform corrosion on the metal surface (Fig. 5a). In contrast, severe pitting was observed on the coupon retrieved from the incubation inoculated with *D. ferrophilus* IS5 in the absence of biocide (Fig. 5b). The frequency and size of pits were comparatively small in incubations where THPS was added as biocide after 3 days of microbiological exposure (Fig. 5c). However, in incubations where GLUT only was added as the biocide, both general corrosion and pitting was observed all over the surface of the metal (Fig. 5d). Treatment with BAC revealed less visual damage to the surface of LC coupon as compared to other biocide combinations tested (Fig. 5e). The GLUT/BAC combination showed overall general corrosion on the surface of the coupon (Fig. 5f) but not the severe pitting that was seen in the case of the incubation of *D. ferrophilus* IS5 with GLUT only (Fig. 5d).

### Electrochemical analysis

**Figure 6:**
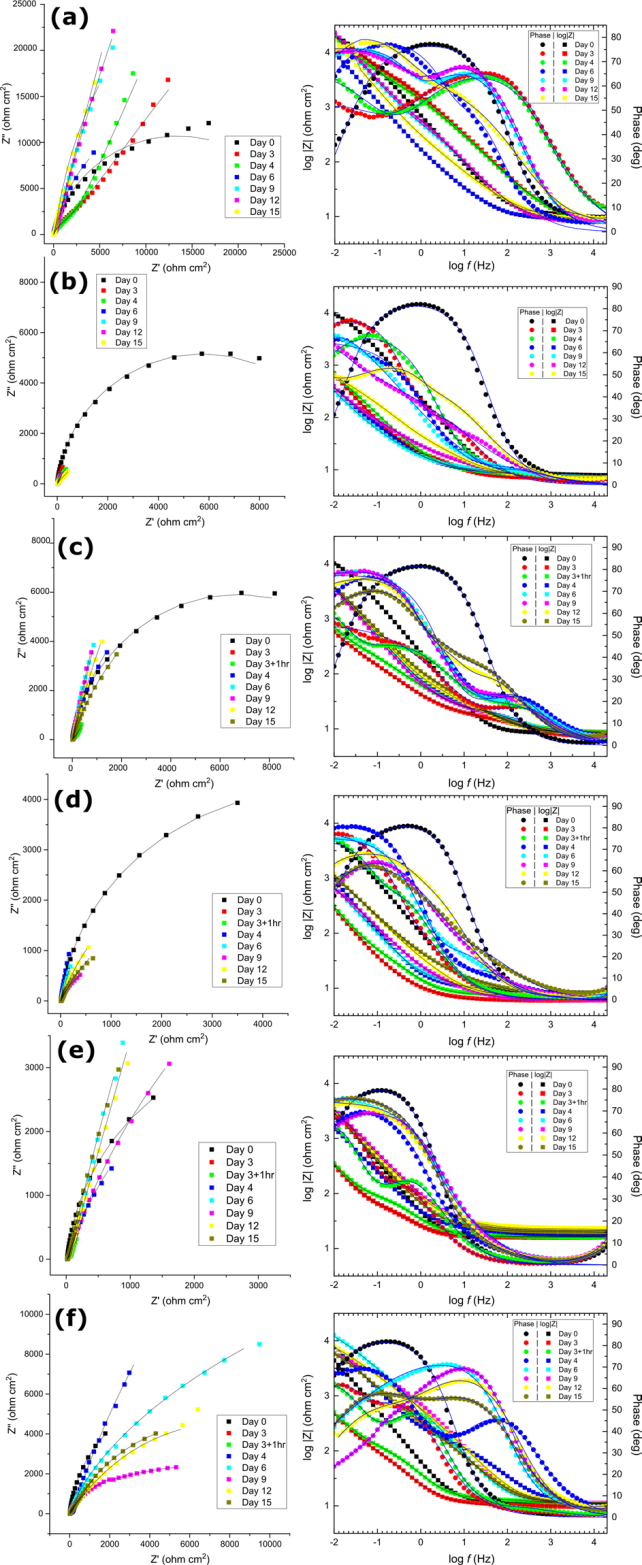
EIS plots (Nyquist plots on left panel, Bode plots on right panel) of all the coupons incubated under various conditions where (**a**) Sterile control, medium only; (**b**) Positive control, medium + IS5; (**b**) IS5 + THPS; (**c**) IS5 + GLUT; (**d**) IS5 + BAC; and (**e**) IS5 + GLUT/BAC. The measurements were taken during the course of the 15-d experiment. Note that biocides were added after 3 days of initial incubation.

LPR and EIS methods are considered non-destructive approaches for measuring corrosion studies *in situ* (in real time)^25^. Figure S2 shows the two equivalent circuits used for the fitting EIS data related to these studies. The results of EIS plots of IS5 with and without incubation with different biocides over a period of 15 days is shown in Fig. 6 where the symbols represent the experimental data and the lines represent fitted data. The Nyquist plots in Fig. 6 (left panel) shows that only one time constant with only one semicircle appears after 15 days of exposure. However, in the Bode plots (Fig. 6, right panel), three time constants can be clearly seen as the incubation progressed post exposure to biocide (e.g., after 3 days of incubation). These can be attributed to biocide and biofilm on the interface. A low maximum phase angle value and the shift in the maximum towards a lower frequency is indicative of active pit growth^8,26^. The absolute magnitude of impedance was also found to be less in the absence of any biocide indicative of high corrosion on the surface of the coupon (Fig. 6). As previously explained by Chen *et al*.^27^, the susceptibility of localized corrosion is proportional to the rate of anodic to cathodic corrosion areas, thus the greater the difference in corrosion rates between cathodic and anodic areas, the more is the metal susceptible to localized corrosion. Bode plots demonstrate the increase in total magnitude of impedance^25^. From these plots, it is evident that the Rp value and maximum phase are significantly increased in the presence of some biocides like BAC (Fig. 6). This indicates the formation of a protective film on the surface of the metal coupon, preventing any kind of microbiological attack, which was visually confirmed with the micrographs (Fig. 5).

**Figure 7:**
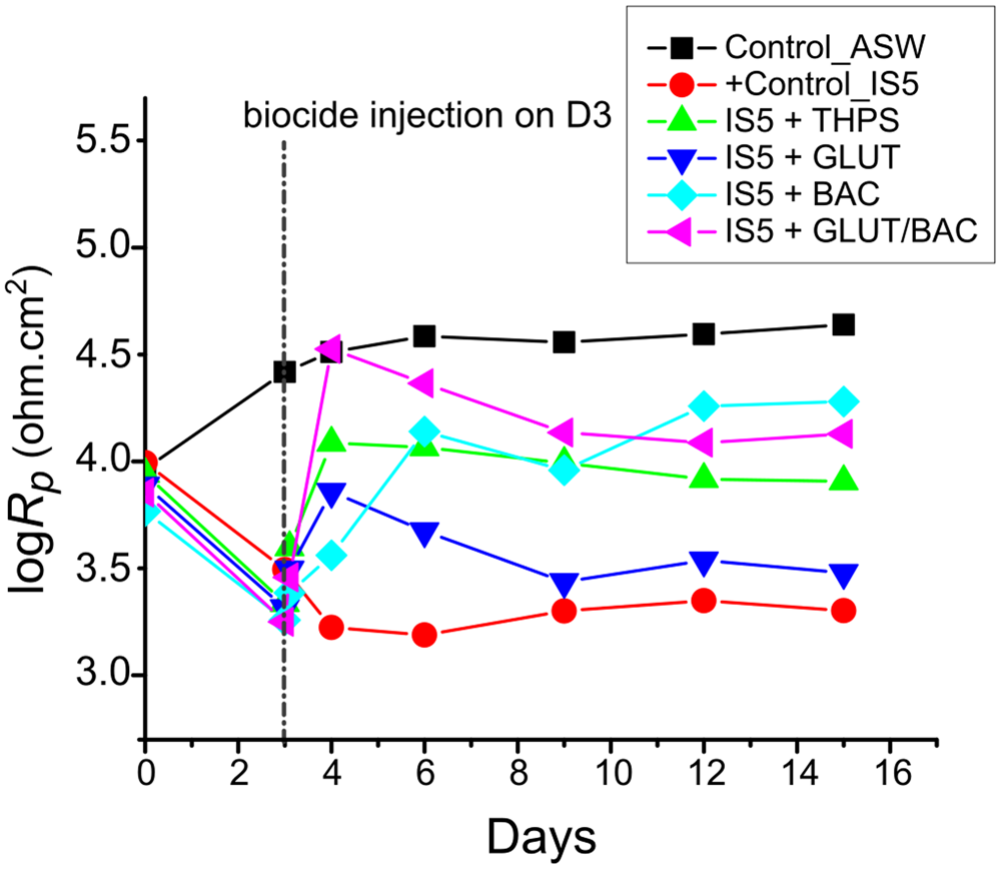
Time dependence of the polarization resistance of all the electrochemical cells during the incubation where the biocide was injected on Day 3 of the experiment except in the sterile control (Control_ASW) and positive control sample containing strain IS5 only (+Control_IS5).

The time dependence of polarization resistance is shown in Fig. 7. The sterile control (ASW medium only) demonstrated a higher Rp throughout the duration of the experiment, indicative of lower corrosion on the coupon surface. In contrast to this, the measurement from the IS5 only incubation showed the lowest Rp which points towards the highest corrosion out of all the different conditions tested. The incubations treated with biocides after 3 days showed a decline in Rp immediately upon the addition of biocides, however the system stabilized by the day 6 till the end of the experiment and higher resistance (that corresponds to lower corrosion) was found in the order of BAC > GLUT/BAC > THPS > GLUT.

**Figure 8:**
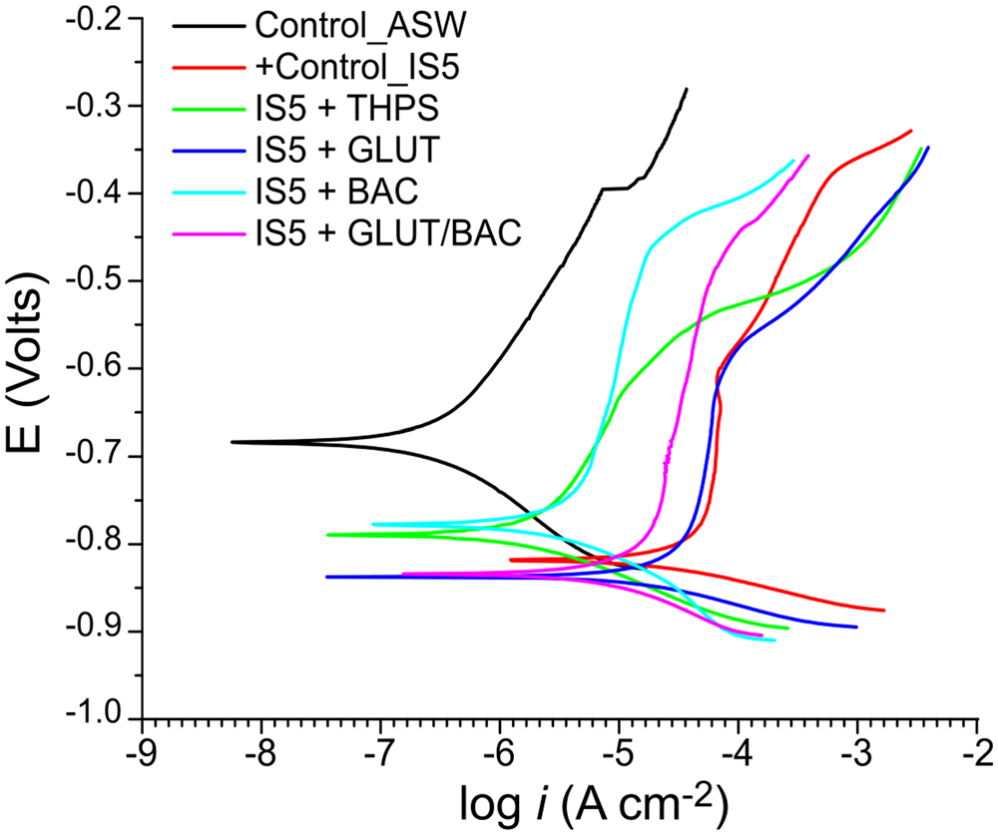
Potentiodynamic polarization plots of all the coupons incubated under various conditions whereby the measurements were taken at the end of the 15-d incubation.

The corrosion current density obtained from Tafel plots was used for determination of corrosion rates as shown in Fig. 8. It can be seen that the Ecorr shifted to the negative direction in the presence *D. ferrophilus* IS5 compared with the control, which indicated that the corrosion activity of steel was enhanced in the presence of SRM. Both the anodic and cathodic reactions were accelerated by *D. ferrophilus* IS5, which further demonstrated that this organism could considerably accelerate LC steel corrosion. After adding biocides, both the anodic and cathodic curves shifted further to the negative direction, leading a lower corrosion current density, as compared to the positive control. These results demonstrated that the use of biocides generally inhibited steel corrosion, but the inhibition effects varied in the presence of various biocides. Out of the four biocides tested, the inhibition effect of BAC against IS5 corrosion was most effective out of the all biocides tested because of the lowest anodic and cathodic reaction rates as well as the smallest corrosion current density. The inhibition effect of GLUT was smallest, as both the anodic and cathodic reaction rates were higher in this case as compared to the other three biocides tested.

Based on the corrosion density values, the corrosion rate of the sterile control incubation was the lowest at 0.005 mm/yr. This highest corrosion rate of 0.49 mm/yr was found in IS5 incubation where no biocide was used to prevent corrosion. In the biocide-amended incubations, the corrosion rate was lowest with the use of THPS at 0.025 mm/yr and BAC at 0.044 mm/yr. This was followed by GLUT/BAC at 0.15 mm/yr and with GLUT at 0.44 mm/yr. Note that the corrosion rate after addition of GLUT is close to that of the IS5 positive control, indicating the ineffectiveness of this biocide under our test conditions. Hence the polarization resistance results align closely with the corrosion rates and surface analysis results observed at the end of the 15-d incubation.

## Discussion

Biofilm formation by microorganisms plays an integral role in corrosion process by either producing a polymer matrix which gets interlinked with the corrosion products and together participate in corrosion, or by altering the microenvironment around the surface of metal which subsequently leads to the formation of reactive chemicals promoting electrochemical corrosion^28–30^. To prevent such biofilm formation on the metal surface, various chemicals including corrosion inhibitors and biocides are used in the oil and gas industry. Many studies in the past have addressed the efficacy of such chemicals in preventing corrosion. For example, Ramesh *et al*.^31^ previously studied the effect of triazole phosphonate based inhibitors and biocide on the corrosion of mild steel in a natural aqueous environment. The efficacy of BAC against carbon dioxide related corrosion and MIC has also been reported^32^. However, despite preventing biofilm growth, these chemicals themselves may chemically react with the surface of metal and promote/reduce corrosion^33,34^. The current study is focused on studying this unique biocide-biofilm interaction on the surface of carbon steel coupons using electrochemical and microscopic methods.

Dinh *et al*.^10^ isolated *D. ferrophilus* strain IS5 from a marine environment and first reported this strain (and others) to be capable of using pure iron as the sole and direct electron donor for its metabolic growth. Since EMIC organisms like strain IS5 have a greater tendency to form biofilms than exist as planktonic forms because of their lithotrophic metabolism, it becomes crucial to study the biofilms of such EMIC organisms in order to find effective biocides which can curtail their growth in a marine environment. Electrical MIC is the main mechanism for these types of microorganisms to directly withdraw electrons from the surface of the metal which may result into high MIC rates of up to 0.7 mm/yr^7^. Weight loss corrosion rates can only provide information related to the general average corrosion rate and may not be a true indicator of corrosion, especially in cases where there is a higher possibility of localized attack^15^. One commonly method for the determination of corrosion is via the use of Tafel slopes where polarization potential (e.g., of generally higher than 100 mV) is applied to the surface of the metal which may lead to irreversible changes on the metal interface of the coupon and hence can only be used once at the end of the incubation. Other non-destructive electrochemical methods are thus important to use in order to monitor the development of corrosion *in situ*. EIS is one such method that can record even minor perturbances that occur on the metal-corrosion product-biofilm interface and give a better estimation of mechanisms taking place at the biofilm-working electrode interface, as the experimental incubation progresses^19,35^. Biofilm monitoring is essential for studying corrosion as biofilms form the primary contact with the surface of the metal where corrosion occurs.

After 15 days of incubation, the coupons incubated under sterile conditions indicated only slight uniform corrosion, while incubations in the presence of strain IS5 showed biofilm and corrosion products spread across the surface of the coupon. More pits were visible in the grain boundaries in the incubation with strain IS5 only as compared to incubations of IS5 with different biocides (with IS5 plus GLUT being the exception). The corrosion crust showed a combination of amorphous and crystalline deposits on the surface of the coupon incubated with IS5, as previously observed by Beese *et al*.^15^. The crystalline crust showed EDX signals of Fe, P, O, and C but no S, while the amorphous areas with SRM biofilm showed Fe, S, O, and C as also previously observed by Beese *et al*.^15^. In incubations/biofilms containing highly corrosive EMIC bacteria like *D. ferrophilus* IS5, LPR may underestimate corrosion rates as these bacteria are more popularly known for causing localized corrosion^15^. Profilometric measurements indicated that the largest pit depth was 30 µm with a diameter of 100 µm after a 15-d incubation of IS5 (Figure S3).

No substantial pits were found in abiotic controls nor in incubations where THPS, BAC, or GLUT/BAC were used for inhibiting microbiological growth. THPS is a quarternary phosphonium compound based biocide that cleaves sulfur-sulfur bonds in the disulfide amino acids of cell walls and also damages the cell membrane. It is also known for its availability to remove iron sulfide deposits^34,36–38^. BAC acts as an amphipathic surfactant molecule that can penetrate the bacterial cell wall resulting in a loss of osmoregulation capacity and cell lysis^32,38,39^. However, due to its amphipathic nature, this biocide may prevent the formation of biofilm and form a thin layer on the surface of the metal coupon preventing it from damage due to microorganisms. BAC has been recently reported to have film-forming corrosion inhibition properties in addition to biocidal properties when it was used to treat MIC by an oil field-derived SRM consortium under flow conditions^40^.

In contrast, incubations with GLUT showed damage all over the surface of the metal along with pitting indicating that this biocide did not protect the surface of the metal from pitting and corrosion damage under our testing conditions. GLUT has reactive electron-accepting functional groups that react with the exposed amine and thiol groups of the membrane proteins of microorganisms, making it a potent cross-linker for amino acids, which can potentially damage cell walls and cause cytoplasmic coagulation^38^^,^^41,42^. However, it is also frequently used as a fixative in biological specimen preparations and this was also seen in the confocal micrographs of the incubations with GLUT, in that a denser biofilm was present of the surface of the coupons. Despite its antibacterial activity, if the cells even after death remain stuck on the surface of metal they may damage the infrastructure in the long run due to the release of extracellular enzymes and hydrogenases by highly corrosive strains. This speculation is supported by some literature studies demonstrating that surface associated redox enzymes, such as hydrogenases and formate dehydrogenase, are sufficient to mediate an apparent electron uptake that enhances iron corrosion in a cell free spent culture medium (e.g., no cells present) of a corrosive microorganism^43^. This study by Deutzmann *et al*.^43^ suggested that the reason for stability of some enzymes is due to their immobilization on solid surfaces or mineral complexes like pyrite (e.g., like the coupons used and mineral corrosion crusts observed in our study). In such a case, the lifetime of enzyme catalytic activity may get extended in crude culture supernatant as compared to purified enzymes, leading to sustained extracellular enzyme-mediated electron transfer. Deutzmann *et al*.^43^ also suggested that electrochemical systems that rely on microbial biofilms forming on electrodes could be particularly prone to lytic release and cumulative sorption of redox active enzymes to an electrode surface. Loss of cell integrity is essential for the enzymes to release, which in our study was likely enabled by the action of GLUT which may have both facilitated cell lysis and immobilized enzymes on the surface of the coupon due to its fixative properties. Interestingly, a detailed study to investigate the electron uptake mechanism of *Desulfovibrio ferrophilus* IS5 was conducted by Deng *et al*.^44,45^ using transcriptomic, bioelectrochemical, and microscopic tools where they showed that this microorganism can extract electrons from reduced sulfide minerals via highly expressed outer membrane cytochromes and extracellular cytochromes under soluble-electron-donor-limited conditions. Since the experiments conducted in this present study were also nutrient-limited and because GLUT can also act as a fixative of the biofilms on the surface of the metal, these fixed biofilms on the surface may have continued to extract electrons from the surface of the working electrode (coupon), resulting in the higher corrosion in the case of IS5 treated with GLUT alone. However, these suppositions require further investigations with *Desulfovibrio ferrophilus* IS5 and other EMIC organisms.

## Conclusions

In the current study, surface analysis and electrochemical methods were used to test the antibacterial efficacy of commonly used biocides in the oil and gas industry against a highly corrosive SRM strain, *D. ferrophilus* IS5. This quick-screening, scaled-down assay is an effective way to select appropriate chemicals for microbiological growth inhibition without compromising the integrity of the infrastructure due to attack by chemical corrosion. *D. ferrophilus* IS5, a model SRM known to utilize electrons directly from metal, can be effectively used for studying MIC as shown in the current study. Non-destructive methods like LPR and EIS are very effective for real time monitoring of MIC. Further, the use of image processing software was also found to be very effective for the semi-quantitative enumeration of live versus damaged cells on the surface of metal and can be effectively adopted as a routine method for cell enumeration/visualization in similar studies. These metal surface analysis and electrochemical tests provided evidence that BAC was most effective biocide in preventing biofilm formation and pitting on the LC steel coupon, compared to other biocides tested in this study, while GLUT alone was the most ineffective under the tested conditions. The study here demonstrated the value of testing biocide efficacies against highly corrosive microorganisms such as *D. ferrophilus* IS5, representing a so-called ‘worse-case’ scenario. Though this and other MIC studies have utilized pure microbial strains to better understand MIC mechanisms and its mitigation^1,2,10,11,17,18,25^, it must be noted that MIC in the natural environment is due to the action of microbial consortia and as such should be the subject of future research.

## Methods

### Culture growth and maintenance

*Desulfovibrio ferrophilus* IS5 was purchased from a culture collection (DSMZ, Braunschweig, Germany). A chemostat connected to a 2-L tank, containing anoxic artificial sea water (ASW) medium and a headspace of N_2_:CO_2_ (80:20) was used for maintaining the culture in active log phase, and served as the inoculum source for the electrochemical experiments described here. The ASW medium consisted of 26 g/L NaCl, 5.6 g/L MgCl_2_, 1.4 g/L CaCl_2_, 2.03 g/L NH_4_Cl, 0.2 g/L KH_2_PO_4_, 0.72 g/L KCl, and was buffered using a CO_2_/bicarbonate buffer. The pH of the medium was adjusted to 7.2–7.4, which did not change during the experiment. The medium was supplemented with 10 mM formate, 1 mM acetate, and 5 mM sulfate as the electron acceptor. Acetate (1 mM) was added to promote biomass as reported in other studies^10,15^. Sodium sulfide (1 mM) was also added to the medium bottle in order to keep the medium anoxic and remove any residual oxygen during the electrochemical cell setup.

### Coupon preparation and electrochemical cell set-up

Low carbon steel coupons (A366), commonly used in marine corrosion studies^3–6^, were used for conducting these experiments where 1 × 1 cm^2^ of the coupon surface was exposed and the other side of the coupon was sealed using epoxy resin leaving one end connected to a processed solid wire (wire-300, VHU 18 GA, NTE Electronics, Inc.). The coupons were polished with silicon carbide papers (from 120 to 1000 grade) using an automated polisher (Buchler Ltd.), then degreased with acetone, washed with deionized water, and dried under stream of N_2_ gas; this was used as the working electrode. Platinum wire (5 × 0.05 cm) was used as a counter electrode, and Ag/AgCl/ in saturated KCl was used as the reference electrode (Fig. 1). The reference electrode was connected to the electrochemical test solution through a lugging capillary filled with a salt bridge consisting of saturated KCl and 1.5% agar. The electrochemical cells were closed with butyl rubbers and flushed with N_2_:CO_2_ (80:20) prior to addition of electrolyte (ASW medium). Six electrochemical combinations (in duplicate, designated a ‘cell set’) were set up. One cell set was designated as the sterile control, containing only ASW medium. All other cell sets were inoculated with 10% inoculum (vol/vol) taken from the chemostat. Once the electrochemical cells were established, they were transferred to a sand bath maintained at 32 °C and these conditions were used for real time monitoring of corrosion using electrochemical methods. The experiment was allowed to proceed for 15 days. On the third day of incubation, four of the cell sets were exposed to a 50-ppm dosage of one of 4 different biocides (THPS, GLUT, BAC, or GLUT/BAC, 50:50, which is a combination of the two biocides in a 1:1 ratio; Table 1; all of which have efficacies at the neutral pH used here^38^), while one incubation cell set was not treated with biocide (positive control). All of the incubations were monitored electrochemically for an additional 12 days.

### Electrochemical measurements

Linear polarization resistance (LPR) measurements were done on all six electrochemical cells over the course of 15 days, with the assumption of Tafel slopes to be 0.2 V^−1^. The polarization resistance (Rp) was calculated after applying a perturbation of ± 10 mV around corrosion potential (Ecorr) with a scan rate of 0.166 mVs^−1^. Electrochemical impedance spectroscopy (EIS) was also conducted on a steady state open circuit potential applied with an amplitude of 10 mV on the electrochemical work station (Model 1280 C, Solartron). As described above, the coupon specimen, platinum wire, and Ag/AgCl/sat KCl were used as working electrode, counter electrode, and the reference electrode, respectively. The frequency for EIS measurements ranged from 2 × 10^4^ Hz to 10^−2^ Hz. The EIS data were collected and fitted using equivalent circuits of the Zsimpwin software. At the end of the 15-d incubation, following EIS, potentiodynamic polarization curves were measured on the working electrode. The potential scan was performed from −200 to +200 mV with respect to the OCP at a sweep rate of 5 mV/sec to derive the cathodic and anodic Tafel slopes *b*_*c*_ and *b*_*a*_. Cview 2 software was used for Tafel fitting. The corrosion current density i_corr_ was calculated using the Stern-Geary equation as previously described^33^.

### Microscopic measurements

#### Confocal measurements and statistical evaluation

Confocal measurements were conducted on all coupons at the end of the incubation period. Staining of the coupons was performed using the Molecular Probes^TM^ Film tracer^TM^ LIVE/DEAD biofilm viability kit. LC steel coupons were washed gently with 0.1 M phosphate buffer followed by staining in the dark room. The SYTO9 stain is a green fluorescent nucleic acid stain used to label all cells (intact and damaged) while propidium iodide was used as a counter stain, which penetrates only bacteria with damaged membranes. The stained specimens were visualized on a confocal laser scanning microscope (CLSM; Olympus-FV-1000).

The raw images obtained post confocal imaging were further processed using the Gwyddion software. This software, used for image processing of confocal micrographs, was used to convert the raw bmp image to an 8-bit image and the fluorescing grains (microbiological cells) in the image were counted using a 10% threshold masking method. This was done in order to remove the background noise in the images and for only selecting the cells present in the micrographs. The resulting statistical data obtained by counting the number of grains directly corresponded to number of cells present on the coupon’s surface while grain coverage corresponded to the percentage of exposed surface area covered with the biofilm. Individual image analysis of each micrograph stained with green (all cells) and red (damaged) stains were used for calculation of relative percentage (average of 3 images) of total and dead/damaged bacteria present on each LC steel coupon’s surface and for relative comparison of efficacy of different biocides used in the present study.

#### Scanning electron microscopy and energy dispersive X-ray measurements

At the end of the experiment, the surfaces of the coupons were gently rinsed with phosphate buffer and incubated with a 2.5% glutaraldehyde fixative solution overnight at 4 °C. The samples were rinsed with 0.1 M phosphate buffer three times for 10 mins every time followed by dehydration using a series of ethanol gradient solutions (30, 40, 50, 70, 80, 90, 95 and 100%). The samples were further dried under a N_2_ gas stream and stored inside a desiccator until visualized^35^. The samples were sputter-coated with platinum and visualized using the JEOL microscope (Japan) at an emission voltage of 10 kV. Energy dispersive X-ray (EDX) measurements were also conducted, yielding qualitative elemental composition.

#### Profilometry and optical microscopy

After the above analyses were conducted, coupons were cleaned using the NACE RP0775–2005 protocol and dried under N_2_ gas stream as previously described^46^. The surface profile of the coupons was subsequently determined using a Zeta 3D stereoscopic microscope. The optical images of the coupons were taken using an optical microscope (NJF-120A, Omax).

## Electronic supplementary material

Supplementary Information

## Data Availability

The datasets generated during and/or analyzed during the current study are available from the corresponding author on reasonable request.
