# Supplementary material for: Effect of selected biocides on microbiologically influenced corrosion caused by Desulfovibrio ferrophilus IS5
Source: Sci Rep. 2018 Nov 9;8:16620. doi: 10.1038/s41598-018-34789-7 (PMC6226443; doi:10.1038/s41598-018-34789-7)
Supplement: Supplementary file 1 — Supplementary Information [file 41598_2018_34789_MOESM1_ESM.pdf]

<u>**Supplementary Information**</u>

Effect of selected biocides on microbiologically influenced corrosion caused by *Desulfovibrio ferrophilus* IS5

Mohita Sharma[1], Hongwei Liu[2], Shiqiang Chen[2,3], Frank Cheng[2], Gerrit Voordouw[1], and Lisa Gieg[1*]

[1]Petroleum Microbiology Research Group, Department of Biological Sciences, and [2]Department of Mechanical and Manufacturing Engineering, University of Calgary, Calgary, Alberta, Canada, T2N 1N4
[3]Present address: Institute of Marine Science and Technology, Shandong University, 72 Binhai Road, Qingdao, 266237, P. R. China

* Corresponding author e-mail: lmgieg@ucalgary.ca

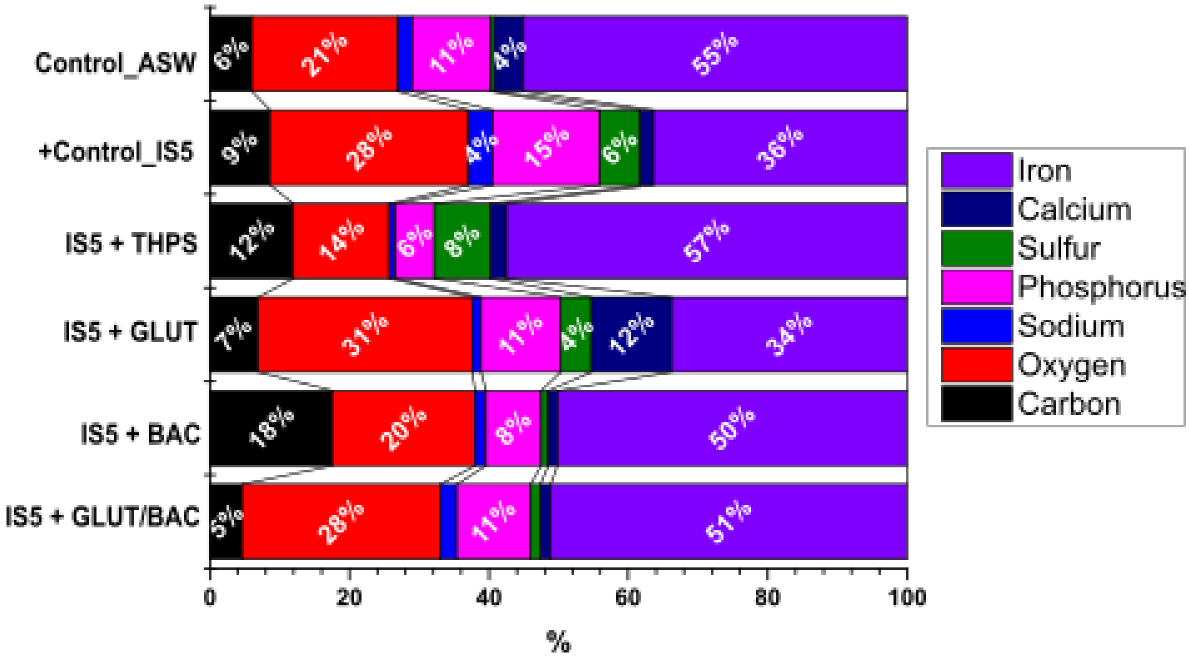

Figure S1: Average elemental composition on the surface of coupons based on weight percentage calculated from energy dispersive X-ray spectroscopy data.

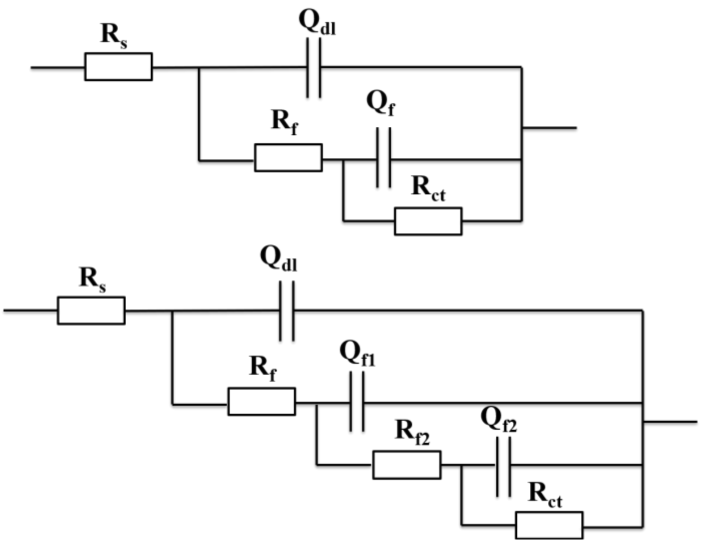

Figure S2: The equivalent circuits used for fitting the EIS data using the ZimSimpWin software.

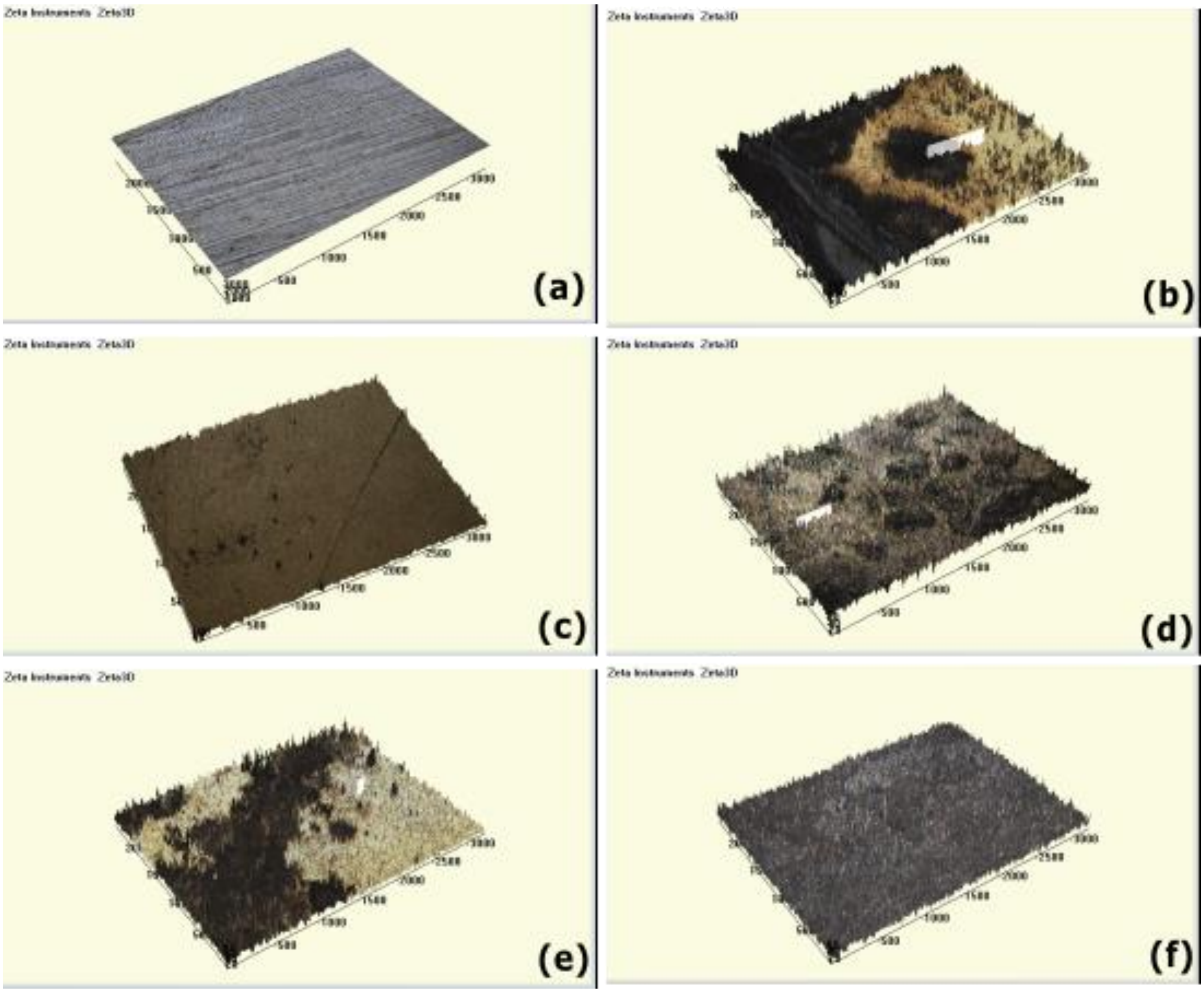

Figure S3: Profilometry images of all the couipon surfaces at the end of the 15-d incubation where (a) Negative Control (ASW medium only), (b) Positive Control (ASW medium + IS5), (c) IS5 + THPS (d) IS5 + GLUT (e) IS5 + BAC (f) IS5 + GLUT/BAC.
